# Integrated housing reconstruction model post-earthquake and tsunamis: Emphasising sustainable construction and local wisdom towards disaster-resilient cities

**DOI:** 10.4102/jamba.v18i1.2011

**Published:** 2026-04-14

**Authors:** Ida Bagus G. Indramanik, Dewa K. Sudarsana, I. Nyoman Y. Astana, Anak G.A. Yana

**Affiliations:** 1 Department of Civil Engineering, Faculty of Engineering, Udayana University, Denpasar, Indonesia

**Keywords:** post-disaster, reconstruction, sustainable construction, local wisdom, disaster resilience city

## Abstract

The 2018 Lombok earthquake caused extensive housing and infrastructure damage in North Lombok Regency, triggering large-scale reconstruction efforts. Post-disaster housing reconstruction (PDHR) is crucial not only for rebuilding damaged physical structures but also for enhancing the resilience of affected communities. However, many reconstruction programmes continue to rely on top-down approaches that neglect sustainability principles and disregard the cultural values inherent in local wisdom (LW). Therefore, this study aimed to develop and test an integrated model that links PDHR, sustainable construction (SC), LW and disaster-resilient cities (DRC). This study adopted a quantitative methodology, with data gathered via a questionnaire survey of 125 disaster-affected residents and analysed by using Structural Equation Modelling–Partial Least Squares in SmartPLS 4.0. The results indicate that PDHR exerts a strong direct effect on DRC (*β* = 1.202, *p* < 0.001) and an indirect effect mediated by SC (*β* = 0.49, *p* < 0.05), confirming the partial mediation mechanism. The results show that reconstruction achieves optimal resilience when sustainability principles are integrated. Conversely, the moderating role of LW was not statistically significant, indicating that LW functions more symbolically than structurally within current reconstruction governance.

**Contribution:**

Theoretically, this study differentiates the structural contribution of SC, evident statistically, from the symbolic contribution of LW, thereby refining existing resilience theory for post-disaster contexts. Practically, the findings underscore the need to redesign institutional mechanisms so that sustainability and LW are not only acknowledged but also formally embedded into reconstruction planning.

## Introduction

Natural hazards such as earthquakes and tsunamis severely disrupt housing systems, critical infrastructure and community livelihoods. Indonesia, as a country, is located within the Pacific Ring of Fire and is among the most disaster-prone countries in the world (Badan Nasional Penanggulangan Bencana [BNPB] 2023). A major disaster was the 2018 Lombok earthquake, which caused 460 deaths, 7.45 trillion rupiah in losses, and 71 962 damaged housing units, comprising 32 129 severe, 20 888 moderate, and 18 945 minor damages, leaving long-lasting social and economic impacts (BNPB 2018).

Post-disaster housing reconstruction (PDHR) is crucial not only for rebuilding damaged physical structures but also for enhancing the resilience of affected communities. The concept of disaster-resilient cities (DRC) emphasises strengthening community capacity to reduce vulnerability to disasters while facilitating faster recovery of social and economic conditions in their aftermath (United Nations Office for Disaster Risk Reduction [UNISDR] 2009, 2017). Recent disaster resilience scholarship further emphasises that resilience is not solely embedded in infrastructure or institutional systems but also is fundamentally rooted in the capacity of individuals and communities to adapt, respond and recover across all phases of disaster management (Tiernana et al. 2019). This concept emphasises the importance of long-term planning that incorporates disaster preparedness, risk management and sustainable urban development.

The reconstruction process faces challenges related to limited resources, an inefficient planning system, and the gap between modern development approaches and local values existing in society (Azmeri et al. 2017; Elsayed 2018). In addition, the absence of effective coordination and comprehensive planning leads to an inefficient planning system, often resulting in overlapping beneficiary data, weak supervision and ineffective relocation management (Asnudin, Ali & Muhtar 2024; Ophiyandri, Amaratunga & Pathirage 2010). Beyond these structural challenges, reconstruction initiatives often fail to adequately integrate cultural and social dimensions, creating a gap between modern development approaches and local values. This gap undermines community participation, cultural acceptance and long-term sustainability (Azmeri et al. 2017; Ophiyandri et al. 2013). The reconstruction process also requires large volumes of construction materials, which are obtained by exploiting nature, which harms nature, and worsens the vulnerability of communities affected by disasters (Barenstein & Pittet 2007; Yasaditama & Sagala 2012). According to Barenstein and Pittet (2007), Elsayed (2018), and Pribadi et al. (2014), the implementation of sustainable construction (SC) in practice should involve efficient resource use, minimal energy consumption, low embodied energy in building materials, reuse and recycling, as well as other mechanisms to achieve effective and efficient utilisation of natural resources in both the short and the long terms.

Local wisdom (LW) plays a crucial role in fostering resilience, as it embodies the community’s traditional knowledge, values and practices for responding to natural hazards (Gunawan 2008; Tucker, Gamage & Wijeyesekera 2014). In the context of disaster management, LW may include the use of environmentally friendly materials and spatial planning that pays attention to risk mitigation. When this approach is neglected, various reconstruction problems arise, such as the rejection, modification or sale of assisted housing by beneficiaries (Ahmed 2011; Pribadi 2020). These previous studies suggest that incorporating LW through community participation fosters ownership and encourages active engagement during reconstruction.

According to global frameworks, the perspectives above are reinforced and can be reintegrated. Indonesia’s Disaster Management Law Number 24 of 2007 and the Sendai Framework for Disaster Risk Reduction 2015–2030 clearly show that sustainability principles and LW are inseparable in the selection and reconstruction processes following disasters to achieve risk reduction and build more resilient communities (NKRI 2007; United Nations Office for Disaster Risk Reduction [UNDRR] 2015). Despite extensive literature on PDHR, SC and LW, previous studies have examined these domains independently rather than integratively. No prior research has empirically tested the combined structural, environmental, and socio-cultural pathways towards resilience, using a Structural Equation Modelling–Partial Least Squares (SEM-PLS) framework. Therefore, this study addresses this gap by developing a PDHR model that incorporates both SC and LW as key elements in achieving DRC.

This model is anticipated to support government and key stakeholders in designing and implementing post-disaster reconstruction strategies that extend beyond damage repair to address long-term vulnerability reduction. Therefore, this study contributes to the development of disaster management theory and practice, particularly in the context of post-earthquake and -tsunami recovery in Indonesia.

This study offers novelty in its approach by simultaneously modelling PDHR, LW and SC, and revealing differential effects, structural versus symbolic, towards disaster-resilient city formation. Furthermore, this study is expected to make a significant contribution to disaster literature and to advance the concept of DRC by integrating principles of sustainability and local values.

On the basis of the literature review, the following hypotheses (H) are developed: (H1) PDHR positively affects DRC, (H2) PDHR positively affects SC, (H3) SC positively affects DRC, (H4) SC mediates the relationship between PDHR and DRC, (H5) LW positively moderates the relationship between PDHR and DRC, (H6) LW positively moderates the relationship between SC and DRC.

## Research methods and design

### Study approach and design

This study employed a deductive quantitative design using SEM-PLS, appropriate for predictive modelling, non-normal data and complex multivariate relationships, whereby the theoretical model was developed based on a comprehensive literature review and then empirically tested by using field data. The design consisted of quantitative and descriptive methods, aiming to explain the relationships among variables through statistical analysis (Hair et al. 2017; Leavy 2017; Yin 2009).

### Study location

**FIGURE 1:**
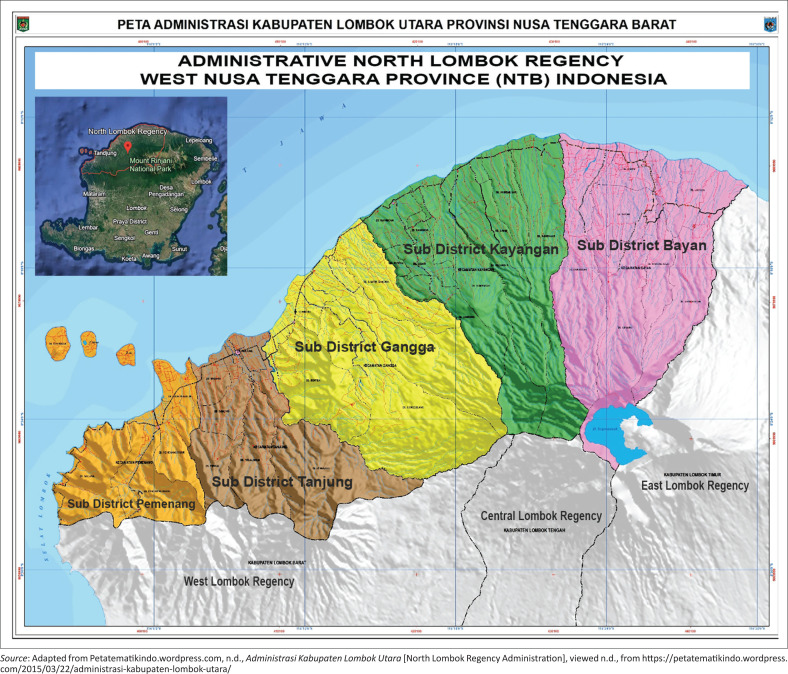
Research study area.

This study was conducted in North Lombok Regency, Lombok, Indonesia, an area that was severely affected by the 2018 earthquake. This location was selected as a result of the significant level of physical damage it experienced and the active role of the community and stakeholders in post-disaster reconstruction efforts. Figure 1 presents a geographic map of the study area.

### Population and sample

In this study, the target population consisted of residents whose houses were damaged by the 2018 Lombok earthquake. Respondents (*n* = 125) were proportionally drawn from five subdistricts, based on damage severity, to improve the representativeness of post-disaster conditions. They were Pamenang (40), Tanjung (40), Gangga (15), Kayangan (15) and Bayan (15).

**FIGURE 2:**
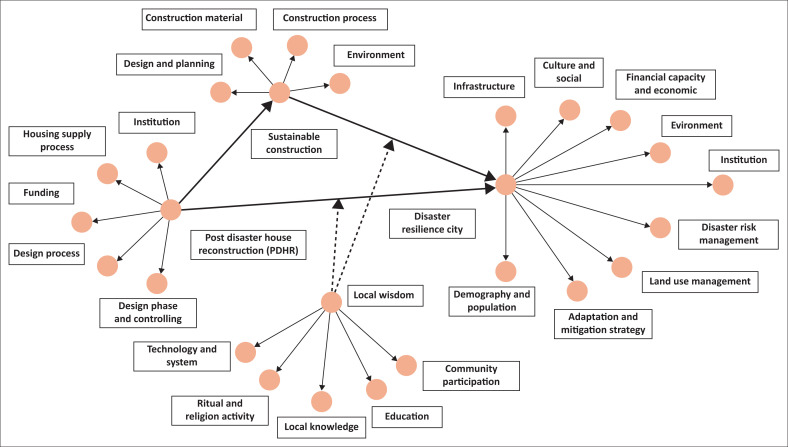
Research model.

The sample size determination referred to Roscoe’s rule of thumb and an a priori power analysis (Hair et al. 2017). Roscoe’s rule of thumb suggests a minimum of 10 times the number of structural paths in multivariate analysis. Given that this study model consists of four latent variables with multiple indicators per construct (Figure 2), the minimum required sample size was 40. Therefore, this study met the requirement with 125 valid responses. For an a priori power analysis using Cohen’s *f*^2^ = 0.15, *α* = 0.05, power = 0.80, and three predictors, the minimum required sample is 77. With 125 respondents, this study meets and exceeds the recommended sample size for PLS-SEM.

### Data collection

A structured questionnaire, developed from established theoretical frameworks and prior studies, was used to collect the data. The dimensions, indicators of each variables of questionnaire shows in Table 1, Table 2, Table 3 and Table 4. The questionnaire used a five-point Likert scale ranging from 1 (strongly disagree) to 5 (strongly agree). A mixed-mode data collection strategy (offline + online) was used to reduce nonresponse bias and ensure accessibility for vulnerable groups affected by the earthquake.

**TABLE 1:** Variables of housing reconstruction after earthquakes and tsunami disasters (X1).

Code	Dimensions	Indicators
X.1.1	Institutions	Involvement of government (BNPB, BPBD and KLU local government), Appropriate government policy on housing reconstruction, Smooth coordination among institutions, Strong leadership.
X.1.2	Housing provision process	Beneficiary identification system, Availability of accurate beneficiary data.
X.1.3	Funding	Availability of financial aid schemes for housing reconstruction, Availability of sufficient aid funds, Equitable distribution of aid fund recipients, Transparency and accountability in aid fund distribution
X.1.4	Design phase	Suitability of house design to occupants’ needs, Reliability of reconstructed house structure in withstanding earthquakes, Use of earthquake-resistant building materials, Adaptation to the local environment through stilt house design, Adaptation to tidal waves and/or tsunamis through elevated foundation buildings, Multi-storey building design as a means of vertical evacuation, Use of corrosion-resistant materials (saltwater) and concrete
X.1.5	Implementation and supervision phase	Coordination and communication between housing providers and beneficiaries, Building materials supply system, Coordination between suppliers and construction implementers, Procurement of construction labour, Project completion timeframe, Quality, safety (K3), environmental supervision and project implementation control

BNPB, Badan Nasional Penanggulangan Bencana [National Disaster Management Agency]; BPBD, Regional Disaster Management Agency; KLU, North Lombok Regency.

**TABLE 2:** Variables of sustainable construction (X2).

Code	Dimensions	Indicators
X.2.1	Planning and design	Use of reconstruction land in accordance with spatial planning, Housing reconstruction with protection of agricultural land, Housing reconstruction with protection of water catchment and river run off areas, Reconstruction location after considering potential disaster risks, Provision of construction materials not through legal means, Planning with involvement of the end-user community, Environmentally friendly and appropriate structural design, Energy-efficient design (daylight lighting, temperature control), Adoption of innovative technologies to reduce disaster risks
X.2.2	Construction materials	Availability of construction material sources, Use of materials suitable to the climate, location, culture and economy, Proper handling of construction debris, Use of debris for elevating reconstructed buildings, Disposal locations for construction debris, Reuse of construction debris in reconstruction, Efficient use of materials, Appropriate material selection, Use of recycled materials, Waste source and disposal management, Availability of required material quantities
X.2.3	Construction process	Construction methods suitable for the location and climate, Construction methods aligned with local context, culture and economy, Environmentally friendly site management, Use of non-polluting materials (water, air and sound)
X.2.4	Environment	Availability of waste treatment, recycling and composting facilities, Pollution prevention, Transport vehicle emissions for material delivery, Use of renewable energy, Energy efficiency, Efficient use of clean water, Management of kitchen and bathroom wastewater

**TABLE 3:** Local wisdom (X3).

Code	Item	Indicators
X.3.1	Technology and systems	Use of local materials, Pyramid or gable-shaped roof, House construction structure with predominant use of reinforced concrete, House construction structure with predominant use of wood, House construction structure with predominant use of bamboo, Interior room layout of the building, Building layout within the yard, Adoption of traditional and/or stilt house construction, Adoption of reinforced concrete house construction, Adoption of traditional building techniques using modern methods, Earthquake-resistant construction techniques
X.3.2	Religious rituals or activities	Religious ritual performance before reconstruction begins, Religious ritual performance after reconstruction is completed, Religious activities to overcome disaster-related trauma during reconstruction
X.3.3	Local knowledge	Early warning of disasters, Natural signs showing disaster occurrence, Evacuation routes
X.3.4	Education	Folktales, Songs and/or poems, Local disaster knowledge included in the school curriculum
X.3.5	Community participation	Mutual cooperation among communities after disasters, Community involvement in the housing reconstruction planning process, Community involvement in the housing reconstruction building process, Community involvement in monitoring housing reconstruction

**TABLE 4:** Disaster resilience city (Y).

Code	Item	Indicators
Y.1	Infrastructure	Buildings and structures designed to withstand earthquakes and tsunamis, Resilient utilities and/or public services, Early Warning System, Location of tsunami evacuation gathering point, Location of tsunami evacuation shelter building
Y.2	Social and culture	Availability and access to basic living services, Community capacity, Community awareness and mitigation efforts, Community involvement
Y.3	Economy and Financial capacity	Government policy and action, Community and surrounding area development, Economic activity diversification, Small business resilience, Jobs and livelihoods, Access to insurance and risk management
Y.4	Environment	Use of ecosystems, Sustainable land-use, Biodiversity and adaptability, Disaster risk reduction based on ecosystems, Community cultural values, Sustainability practices.
Y.5	Institution	Collaboration between Institutions, Good Governance
Y.6	Disaster risk management	Identification and mapping of risks, Understanding of vulnerabilities, Implementation of risk reduction measures, Preparation for effective response and recovery
Y.7	Land-use management	Risk mapping, Disaster zoning, Ecosystem conservation, Community Involvement.
Y.8	Mitigation and adaptation strategies	Implementation of effective disaster mitigation strategies, Development and implementation of disaster adaptation plans, Capacity building of communities in disaster response, Utilisation of technology and innovation to enhance disaster resilience.
Y.9	Population and demographics	Low population density, Diverse population structure (age, gender and other demographic characteristics), Good human resource capacity, Adequate community awareness and education

In addition to the survey, semi-structured interviews were conducted with village heads, traditional leaders, disaster survivors, local government officials and other stakeholders. These interviews served to validate and enrich the quantitative results.

Data collection was carried out from March 2024 to July 2024. All 125 questionnaires distributed to respondents were returned with complete responses. The data were then tabulated and analysed according to the planned procedures.

A pilot test (*n* = 30) was conducted to assess item validity and reliability. Items with inadequate loading (< 0.50) were removed following Hair et al. (2017), improving construct validity before full deployment. After confirming the instrument’s validity and reliability, the remaining 95 respondents were surveyed. The final dataset comprising 125 responses was analysed by using SEM-PLS to test the hypotheses and develop the integrated reconstruction model.

### Data analysis method

The collected data were analysed by using the SEM-PLS method with the assistance of SmartPLS 4.0. Software. Structural Equation Modelling–Partial Least Squares SmartPLS 4 (SmartPLS GmbH, Oststeinbek, Schleswig-Holstein, Germany) was selected as a result of its suitability for: (1) exploratory and predictive modelling, (2) non-normal data structures, (3) complex models with mediators and moderators and (4) relatively small samples (< 250). Bootstrapping (5000 subsamples) was applied for hypothesis testing. All constructs were specified as reflective indicators because changes in the latent variable are theoretically expected to cause changes in all associated indicators (Hair et al. 2019).

The analysis was conducted in two main stages, including (1) evaluation of the measurement model (outer model) to assess indicator reliability, convergent and discriminant validity and multicollinearity, and (2) evaluation of the structural model (inner model) to test path coefficients, *R*^2^, *f*^2^ and *Q*^2^ values. Validity and reliability checks were conducted in two phases, such as a pilot test with 30 respondents, followed by full analysis using the complete sample (*n* = 125).

### Variables and indicators

The model included four latent variables measured reflectively: PDHR (X1), SC (X2), LW (X3) and Disaster-Resilient City (Y). Each construct was modelled reflectively. Indicators with factor loadings < 0.50 were excluded from further analysis to ensure convergent validity in accordance with. The criteria for indicators that were excluded refer to Hair et al. (2017). Final retained indicators are presented in Table 5, Table 6, Table 7 and Table 8:

- Post-Disaster Housing Reconstruction (X1)

**TABLE 5:** Results of convergent validity test for the post-disaster housing reconstruction variable (X1).

Variable	AVE	Outer loading
Institution	0.781	0.884
Funding	0.583	0.748
Design phase	1.000	1.000
Construction phase and controlling	0.604	0.769

AVE, average variance extracted.

**TABLE 6:** Results of the convergent validity test for the sustainable construction variable (X2).

Variable	AVE	Outer loading
Planning	0.666	0.815
Construction material	0.630	0.725
Construction process	0.745	0.862
Environment	0.700	0.836

AVE, average variance extracted.

**TABLE 7:** Results of the convergent validity test for the local wisdom variable (X3).

Variable	AVE	Outer loading
Technology_ and system	0.704	0.839
Ritual and/or religion activity	0.744	0.860
Local knowledge	0.650	0.806
Education	1.000	1.000
Community_ participation	0.786	0.887

AVE, average variance extracted.

**TABLE 8:** Results of the convergent validity test for the disaster-resilient city variable (Y).

Variable	AVE	Outer loading
Society and culture	0.607	0.768
Economic and financial capacity	0.901	0.951
Environment	0.613	0.783
Institutions	0.777	0.881
Disaster risk management	0.566	0.735
Land-use management	1.000	1.000
Mitigation and adaptation strategy	0.619	0.786

AVE, average variance extracted.

This variable shows efforts to rebuild housing following the 2018 earthquake in North Lombok. It consists of five reflective dimensions, including institutional aspects, housing provision process, funding, planning and construction implementation.

Each dimension consists of reflective indicators. These indicators refer to Ahmed (2011), Karunasena and Rameezdeen (2010), Li (2019), and Pamidimukkala, Kermanshachi and Safapour (2020):

- Sustainable Construction (X2)

This variable captures the application of environmentally friendly and socially responsible construction practices in the context of post-disaster reconstruction. The dimensions include planning and/or design, construction materials, construction process and environmental impact. Each dimension consists of reflective indicators. These indicators refer to Aarseth et al. (2017), Halliday (2018), and Mushtaha and Alaloul (2025):

- Local wisdom (X3)

Local wisdom represents the knowledge and cultural practices of communities relevant to disaster resilience. Its dimensions include traditional technologies and/or systems, religious rituals, local knowledge, education and community participation. These indicators refer to Kusumasari and Alam (2012), Ophiyandri et al. (2010), and Pribadi et al. (2014):

- Disaster resilience city (Y)

This variable reflects a city’s resilience to earthquakes and tsunamis. It consists of nine dimensions: infrastructure, socio-cultural aspects, economic capacity, environmental sustainability, institutional strength, disaster risk management, land governance, mitigation and adaptation strategies, and demographics. Each dimension consists of reflective indicators. These indicators refer to Al-Humaiqani and Al-Ghamdi (2022), De Iuliis, Kammouh and Cimellaro (2022), Hein and Schubert (2021), and Ribeiro and Pena Jardim Gonçalves (2019).

## Results

This section outlines the findings of the SEM-PLS-based data analysis conducted with SmartPLS 4.0. The analysis was conducted in two main stages, namely, the evaluation of the measurement model (outer model) and the evaluation of the structural model (inner model):

### Measurement model evaluation (outer model)

This study conducted an evaluation of the measurement model to ensure the validity and reliability of the latent constructs.

The validity was assessed based on convergent validity and discriminant validity values.

#### Validity test

**Convergent validity test:** The convergent validity was assessed through Loading Factor (LF) values and the Average Variance Extracted (AVE) values.

Table 5, Table 6, Table 7 and Table 8 show that the LF values are above 0.50 (LF > 0.5), following the recommendations of Fornell and Larcker (Hair et al. 2017). These results show that all measurement indicators have met the required convergent validity, and each indicator accurately measures the intended variables.

Therefore, it can be concluded that these indicators are appropriate to be used in forming their respective reflective constructs. Indicators with LF < 0.50 from the pilot test were removed before the final model estimation, ensuring convergent validity and improving construct reliability (Hair et al. 2017).

Table 5, Table 6, Table 7 and Table 8 also show that the AVE values of the constructs meet the required standard, namely AVE > 0.5. All constructs achieved AVE > 0.50.

More than half of the variance in the indicators is explained by their respective latent variables. This confirms satisfactory convergent validity.

From the results of the analysis, it was obtained that there were sub-variables that had an LF value of less than 0.50 (LF < 0.50) and more than 0.50 (LF > 0.50).

According to Fornell and Larcker’s criteria (Hair et al. 2017), only sub-variables that have an LF value of > 0.50 are included in the subsequent calculation, which means that these results show that all measurement indicators have met the required convergent validity, and each indicator accurately measures the intended variables. Therefore, it can be concluded that these indicators are appropriate to be used in forming their respective reflective constructs (Table 9).

**TABLE 9:** Fornell–Larcker criterion.

Variable	X2	X3	Y	X1
**X2**	0.789	-	-	-
**X3**	0.659	0.797	-	-
**Y**	0.552	0.673	0.765	-
**X1**	0.505	0.665	0.605	0.799

The results of the analysis show that the AVE values of the constructs meet the required standard, namely AVE > 0.50. This outcome shows that the constructs in this study are capable of measuring their associated latent variables.

**Discriminant validity:** Discriminant validity was evaluated by using the Fornell–Larcker criterion and cross-loadings. The square roots of AVE exceeded inter-construct correlations, and all indicators met the required thresholds (cross-loadings > 0.70; AVE > 0.50), confirming that PDHR, SC, LW and DRC are empirically distinct and that discriminant validity is achieved.

##### Reliability testing

Reliability was assessed by using PLS-based internal consistency with Composite Reliability (CR) and Cronbach’s alpha ≥ 0.60. As shown in Table 10, all latent variables meet these criteria, and the Cronbach’s alpha (> 0.60) and AVE (> 0.50) values confirm reliable and stable measurements across PDHR, SC, LW, and DRC.

**TABLE 10:** Reliability test results.

Variable	Cronbach’s alpha	CR (rho_a)	CR (rho_c)	AVE
**X2**	0.949	0.951	0.955	0.623
**X3**	0.952	0.954	0.958	0.636
**Y**	0.940	0.942	0.948	0.585
**X1**	0.929	0.929	0.941	0.639

CR, composite reliability; AVE, average variance extracted.

The results of the analysis showed that the overall value of Cronbach’s alpha > 0.60 and AVE > 0.50, which confirmed the reliability of internal consistency as well as the stability of measurements across the dimensions of PDHR, SC, LW and DRC. The evaluation of outer loading also showed that all indicators met the required value criteria.

### Structural model assessment

This evaluation stage was carried out to confirm the theoretical model as outlined in the structural study model.

#### *R*-Square (*R*^2^) value

The model explains 69.6% of the variance in DRC, which is considered a strong explanatory level in PLS-SEM (Hair et al. 2019). This result indicates that PDHR, SC and LW collectively form a robust explanatory framework for disaster resilience.

The categories are Weak (*R*^2^ < 0.25), Fair (0.25 ≤ *R*^2^ < 0.50), Good (0.50 ≤ *R*^2^ < 0.75) and Excellent (*R*^2^ ≥ 0.75).

Based on the results shown in Table 11, the constructs are in the good category, with *T*-statistic values exceeding 1.96 and *p*-values equal to 0.000, showing that all relationships are statistically significant.

**TABLE 11:** *R*-square (*R*^2^) value.

Variable	Original sample (O)	Sample mean (M)	Standard deviation (s.d.)	*T*-statistics (|O/s.d.|)	*P*-value
Disaster resilience city	0.696	0.703	0.053	13.109	0.000

s.d., standard deviation.

#### Goodness of fit

Goodness of fit is used to evaluate the overall suitability of the model by integrating the measurement model (outer model) and the structural model (inner model). The results of the analysis showed a goodness-of-fit value of 0.659, which was included in the *good* category (> 0.36), thus showing an excellent level of model suitability. These findings confirm that the integrated model is able to adequately represent the measurement and structural components and is suitable for predictive and explanatory purposes in post-disaster studies.

#### Conclusion of the outer model and structural model evaluation

The results of the outer model evaluation show that all constructs used are valid and reliable, allowing for further analysis of the study model. Based on the structural model evaluation results, the proposed model is appropriate, and thereby hypothesis testing can be conducted.

### Hypothesis testing

**FIGURE 3:**
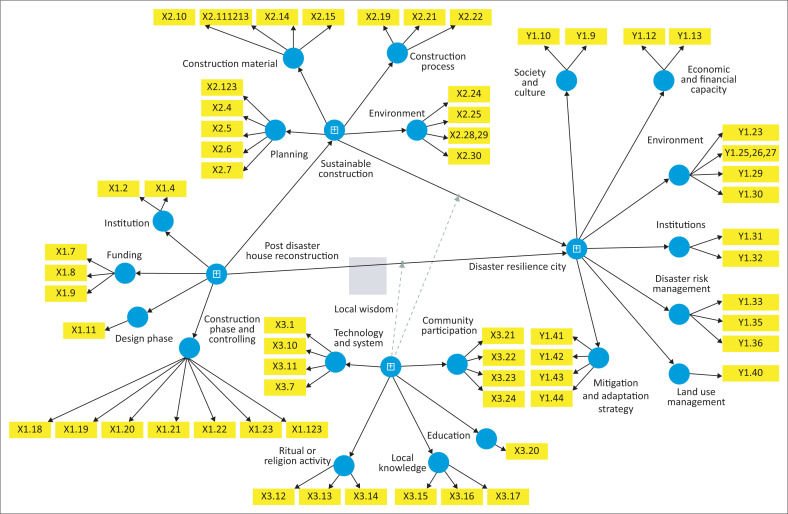
SEM-PLS Structural model of post-disaster housing reconstruction, sustainable construction, and local wisdom toward disaster-resilient city.

The significance of the relationships between latent variables was examined through hypothesis testing. Hypothesis testing was calculated based on the path coefficient results and model significance, as shown by the *p*-value. A path coefficient is considered significant only if the *p*-value is less than 0.05. Table 12 and Figure 3 present the *p*-values of the relationships between variables in this study.

**TABLE 12:** Results of Hypothesis Testing (Bootstrapping).

Variable	Original sample (O)	Sample mean (M)	Standard deviation (s.d.)	*T*-statistics (|O/s.d.|)	*P*-value
Post disaster house reconstruction → Disaster resilience city	1.202	1.201	0.199	6.053	0.000
Post disaster house reconstruction → Sustainable construction	0.907	0.907	0.017	54.382	0.000
Sustainable construction → Disaster resilience city	0.541	0.537	0.166	3.260	0.001
Local wisdom → Disaster resilience city	−0.998	−0.995	0.285	3.503	0.000
Local wisdom × Post disaster house reconstruction → Disaster resilience city	−0.038	−0.034	0.155	0.243	0.808
Local wisdom × Sustainable construction → Disaster resilience city	−0.102	−0.108	0.161	0.633	0.527

s.d., standard deviation.

Table 12 shows that the PDHR → DRC line had a positive and significant effect (*β* = 1.202; *t* = 6.053; *p* = 0.000), so that H1 was accepted, while the PDHR → SC line was also significant (*β* = 0.907; *t* = 54.382; *p* = 0.000), so that H2 was accepted, and the SC → DRC line had a significant positive effect (*β* = 0.541; *t* = 3.260; *p* = 0.001), so that H3 was accepted. Mediation analysis showed that the direct influence of PDHR → DRC remained significant (*β* = 1.202; *p* = 0.000) and that the indirect influence through SC was also significant (*β*_indirect ≈ 0.49), so H4 was accepted and confirmed partial mediation. In contrast, the moderation effects of LW × PDHR → DRC (*β* = –0.038; *t* = 0.243; *p* = 0.808) and LW × SC → DRC (*β* = –0.102; *t* = 0.633; *p* = 0.527) were not significant, so H5 and H6 were rejected, suggesting that LW has not been adequately institutionalised to moderate the relationship on urban resilience levels.

## Discussion

The strong effect of PDHR on DRC (*β* = 1.202, *p* < 0.001) confirms that reconstruction is a structural determinant of resilience. Valid indicators of PDHR, such as institutional coordination (X1.1.3), transparent beneficiary identification (X1.2.1), and earthquake-resistant structural design (X1.4.2), directly contribute to restoring functionality, reducing exposure and strengthening community security, three core components of urban resilience.

The significant effect of PDHR on SC (*β* = 0.907, *p* < 0.001) indicates that reconstruction decisions, particularly in planning and design (X2.1), selection of local and low-carbon materials (X2.2), and environmentally responsible construction management (X2.3), shape the extent to which sustainability is embedded in post-disaster rebuilding. This finding is in line with (Tucker et al. 2014; Yasaditama & Sagala 2012), who reported that sustainable principles are used in reducing disaster vulnerability. These include the use of environmentally friendly materials (e.g. the use of red bricks, the use of building debris for the elevation of buildings and yards), energy resource efficiency and waste minimisation. The reconstruction phase typically demands substantial materials and energy, and neglecting sustainability considerations can result in environmental degradation. Therefore, integrating SC into reconstruction is not only environmentally important but also a strategy to enhance community resilience in the long-term and reduce vulnerability in North Lombok. The integration of SC into PDHR ensures housing safety while maintaining ecological balance, which is crucial for this earthquake- and tsunami-prone island region.

Reconstruction requires substantial materials and energy; therefore, integrating SC into PDHR is essential to prevent environmental degradation, enhance long-term community resilience and maintain ecological balance in the earthquake- and tsunami-prone region of North Lombok.

SC significantly improves DRC (*β* = 0.541, *p* = 0.001). Indicators such as sustainable land-use alignment (X2.1.123), energy-efficient design (X2.1.5) and environmental protection measures (X2.4.x) reduce long-term ecological risks and enhance recovery capacity.

This observation is in line with results from Barenstein and Pittet (2007), Capell and Ahmed (2021), and Tucker et al. (2014), which emphasise that the use of sustainable materials and environmentally friendly designs significantly reduces environmental impacts and enhances long-term adaptive capacity. In the post-disaster context, integrating SC ensures that the reconstruction process not only restores damage but also improves community preparedness for future disasters.

In this study, the analysis shows that SC partially mediates the relationship between PDHR and DRC. The direct effect of PDHR on DRC remains significant (*β* = 1.202, *p* < 0.001), and the indirect effect through SC is also significant (*β*_indirect ≈ 0.49, *p* < 0.05).

This result shows that although reconstruction itself enhances resilience, the integration of SC practices within reconstruction further strengthens this relationship. The mediating role of SC confirms that reconstruction programmes will achieve optimal resilience outcomes when adopting SC principles. Post-disaster housing reconstruction initiates structural recovery, but SC ensures that such recovery is durable, environmentally sound and future-proof. This result is consistent with Tucker et al. (2014), who emphasised that environmentally friendly designs and sustainable materials enhance adaptive capacity in post-disaster housing. Similarly, Barenstein and Pittet (2007) highlighted that conventional reinforced concrete approaches increase environmental burdens, while sustainable and locally appropriate practices reduce vulnerability and support resilience. Furthermore, Capell and Ahmed (2021) reported that the adoption of SC principles ensures reconstruction outcomes that are socially and environmentally sustainable, thereby maximising resilience benefits. In the Indonesian context, Pribadi et al. (2014) also reported that PDHR offers an opportunity to ‘build back better’ by integrating disaster risk reduction and sustainability measures into reconstruction programmes. In addition, these studies confirm the mediating role of SC, showing that optimal resilience outcomes are achieved when reconstruction initiatives embed sustainability as a core principle.

Contrary to the hypothesis, LW does not significantly moderate the relationship between PDHR and DRC (*β* = –0.038, *p* = 0.808) nor between SC and DRC (*β* = –0.102, *p* = 0.527). This finding shows that, within the context of this study, LW does not statistically strengthen or weaken these relationships.

The results of the statistical analysis show that the interaction between LW and PDHR is not significant in predicting urban resilience.

Indicators of LW, such as traditional technology and system in construction techniques (X3.1), spatial layout (X3.1.5), ritual practices (X3.2) and local hazard knowledge (X3.3), operate dominantly at the household and community scale. Meanwhile, DRC is a city-level construct driven by infrastructure governance, institutional arrangements and systemic risk management. This scale mismatch weakens LW’s statistical influence.

These findings are consistent with Gaillard-Waipapa et al. (2022), who argue that local knowledge remains impactful only when formally integrated into institutional decision-making. In North Lombok, LW contributed symbolically (cultural identity and rituals) but was not embedded into PDHR’s regulatory and engineering frameworks, reducing its moderating potential.

In many cases, housing reconstruction programmes are still dominated by a top-down approach with an emphasis on efficiency, standardisation and budget (Azmeri et al. 2017; Barenstein & Pittet 2007; Ophiyandri et al. 2010). As a result, LW functions more symbolically than practically, and its influence remains insufficient to moderate the impact of reconstruction on the development of DRC.

This unexpected result makes an important theoretical contribution by challenging the assumption that LW always reinforces recovery outcomes. Previous studies have emphasised the role of community-based knowledge in building resilience (Ahmed 2011; Gunawan 2008). But the results of this study show a difference in scale. Local wisdom operates at the household and community levels, while urban resilience is shaped through governance structures, infrastructure systems and institutional capacity (Elsayed 2018). An investigation by Kinanti, Suwena and Sudiarna (2019) supports these results by explaining why LW does not moderate the relationship between PDHR and SC and DRC. The case in Bayan Village, North Lombok, shows that the community rejects earthquake-resistant house innovation of the Healthy Simple Instant House (Rumah Instan Sederhana Sehat [RISHA]) promoted by the government. The rejection was rooted in cultural and spiritual beliefs. This observation illustrates how LW can even counteract top-down reconstruction efforts.

In addition, the top-down reconstruction policy further weakens LW integration. As reported by Barenstein and Pittet (2007), Capell and Ahmed (2021), the government emphasises technological innovation more than two-way communication with the community. As a result, local values and practices are not accommodated in the PDHR programme, thereby LW loses its potential as a moderation factor. The forms of resistance of the Bayan people, ranging from traditional deliberations, the destruction of modern elements in the Bayan Beleq Ancient Mosque, to the construction of houses independently, show that LW can be a means of resistance to innovations that are considered not in accordance with local norms, rather than strengthening the relationship among PDHR and SC and the DRC (Kinanti et al. 2019).

The interaction among dimensions reveals a coherent pattern: planning and design quality (PDHR and SC indicators), material sustainability and environmental management jointly reinforce systemic resilience. However, LW contributes primarily to social cohesion and risk awareness rather than structural or institutional resilience.

Theoretically, this study proposes an integrated model that links reconstruction, sustainability and LW in the formation of urban resilience, while affirming the mediating role of SC and the complexity of the position of LW.

Practically, policymakers and reconstruction practitioners need to integrate sustainability principles into the PDHR and encourage meaningful community participation. The integration of LW will ensure social legitimacy, long-term functionality and community acceptance, so it needs to be prioritised through policies, training programmes and supportive funding mechanisms.

## Conclusion

This study confirms that PDHR has a strong direct influence on the formation of DRC, emphasising the importance of institutional coordination, structural design and housing provision systems in shaping urban resilience. The results also indicate that SC significantly improves disaster resilience and partially mediates the relationship between reconstruction and resilience, indicating that reconstruction will make the most effective contribution when supported by sustainability-oriented practices in planning, environmental management and material selection.

The findings of the study indicate that LW is still playing a primarily symbolic role in post-disaster reconstruction, with the main contribution to strengthening cultural identity and social cohesion, rather than to structural resilience. To go beyond such symbolic roles, LW needs to be formally mandated within the framework of reconstruction planning and governance, so that communities are not only encouraged but also required to integrate their local knowledge into the reconstruction process.

Therefore, this study concludes that an integrated PDHR model by effectively incorporating SC and acknowledging the crucial practical role of LW can lead to more inclusive, safe, sustainable and disaster-resilient reconstruction.

Theoretically, this study refines resilience scholarship by distinguishing between structural determinants (PDHR and SC) and symbolic socio-cultural determinants (LW). The results advance understanding of how reconstruction and sustainability jointly shape resilience in post-disaster contexts.

Practically, these results highlight the need for governments to institutionalise sustainability standards in reconstruction programmes and to integrate local socio-cultural insights into planning processes. Embedding LW into regulatory, design and community engagement frameworks may increase its structural relevance in future disasters.

### Recommendations

Overall, the integrated model reveals that reconstruction and sustainability are foundational to resilience, while LW requires stronger institutional integration to exert a measurable influence. Future studies should explore hybrid models combining socio-technical indicators and longitudinal designs to capture evolving resilience trajectories.

### Limitations

The limitations of the research include the limited coverage of North Lombok, the use of the Likert scale that does not fully capture the socio-cultural dimension, and the explicit inclusion of policy and governance variables.
